# Advances and Challenges in Managing Hepatitis D Virus: Evolving Strategies

**DOI:** 10.1007/s11901-024-00643-w

**Published:** 2024-02-01

**Authors:** Harish Gopalakrishna, Maria Mironova, Harel Dahari, Christopher Koh, Theo Heller

**Affiliations:** 1 Liver Disease Branch, National Institute of Diabetes and Digestive and Kidney Diseases, National Institutes of Health, Bethesda, MD, USA; 2 The Program for Experimental & Theoretical Modeling, Division of Hepatology, Department of Medicine, Stritch School of Medicine, Loyola University Chicago, Maywood, IL, USA; 3 Translational Hepatology Section, National Institute of Diabetes and Digestive and Kidney Diseases, National Institutes of Health, 10 Center Drive, Building 10, Room 4-5722, Bethesda, MD 20892-1800, USA

**Keywords:** Hepatitis D virus, Delta hepatitis, Hepatitis B, Antiviral therapy, Lonafarnib, Bulevirtide

## Abstract

**Purpose of Review:**

Hepatitis D Virus (HDV), although a small defective virus, poses a substantial public health challenge due to lack of awareness, underrecognized prevalence, and limited treatment options. Universal HDV screening within hepatitis B virus (HBV) cohorts is essential to address this issue. Despite its aggressive nature, effective HDV therapies have remained elusive for over four decades.

**Recent Findings:**

Advances in understanding HDV’s biology and clinical behavior offer potential therapeutic breakthroughs, fostering optimism. As insights grow, effective and targeted therapies are being developed to improve HDV management.

**Summary:**

This review delves into HDV’s intricate structure and biology, highlighting formidable hurdles in antiviral development. It emphasizes the importance of widespread screening, exploring noninvasive diagnostics, and examining current and emerging innovative therapeutic strategies. Moreover, the review explores models for monitoring treatment response. In essence, this review simplifies the complexities of effectively combating HDV.

## Introduction

Hepatitis D Virus (HDV), also known as hepatitis delta virus, is the smallest yet one of the most potent pathogens, presenting a formidable global public health threat. HDV infection occurs in individuals who are co-infected with hepatitis B virus (HBV), resulting in an aggressive form of viral hepatitis [1]. The combination of HDV and HBV accelerates disease progression, with approximately 70–80% of patients developing cirrhosis within few decades of infection [2].

Recent estimates reveal that HDV impacts 5% of HBV-infected individuals, affecting around 12 million people globally [3•]. However, true prevalence is likely higher due to underutilized screening methods [4, 5]. Despite its substantial impact, clear monitoring guidelines for diagnosed cases are lacking. Advances in serological testing and non-invasive screening strategies are revolutionizing HDV management, reshaping the field [6].

Despite its discovery over four decades ago, there are no Food and Drug Administration (FDA)–approved therapies specifically targeting HDV, leaving patients with limited treatment options. Interferon-alpha is often used based on professional liver societies’ recommendations, but its success rates are generally modest [7]. Though bulevirtide, authorized in Europe in July 2020, marked a breakthrough cure for HDV, a potential gateway to HDV resolution remains elusive [8••].

With complex epidemiology, a rapid disease trajectory, and scarce treatment options, precise diagnosis and disease stratification assume paramount importance. This review aims to shed light on key aspects of managing HDV. It discusses about the intricate structure and biology of HDV, uncovering substantial challenges in antiviral development. This review highlights the importance of widespread screening, explores noninvasive diagnostics and current and emerging innovative therapeutic strategies.

### Hepatitis D Virus: a Unique and Complex Pathogen

**Fig. 1:**
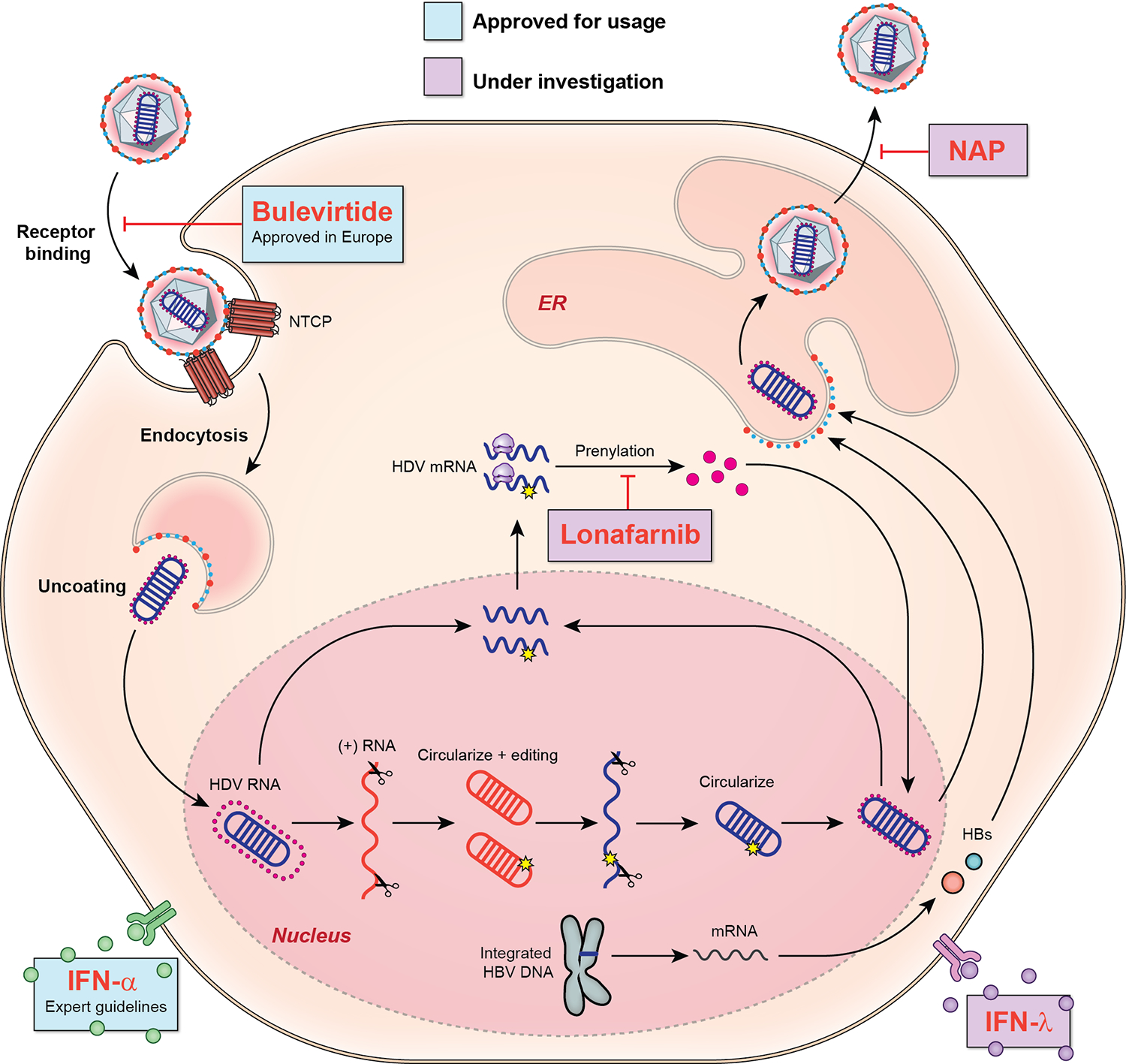
HDV life cycle and sites of drug targets. HDV virion attaches to the hepatocyte via the sodium taurocholate cotransporting peptide (NTCP). HDV ribonucleoprotein (RNP) is translocated to nucleus. In the nucleus, HDV genome replication occurs by rolling-circle mechanism. HDV antigenome is transported out of the nucleus to the endoplasmic reticulum (ER). New HDV RNP associates with HBV envelop proteins and assemble into HDV virions. Completed HDV virions are released from the hepatocyte. Drugs approved for usage—as per expert guideline: interferon alpha (IFN-α), conditional approval in Europe: bulevirtide. Drugs under investigation—nucleic acid polymer (NAP), lonafarnib and interferon lambda (IFN-λ)

HDV, a satellite virus reliant on HBV for its life cycle (Fig. 1), is categorized as a “defective,” or “incomplete virus” [9]. The structure of HDV consists of an enveloped particle, 35–37 nm in size, harboring the delta antigen and a circular single-stranded RNA genome covered by the hepatitis B surface antigen (HBsAg) lipoprotein from HBV [10]. This resemblance enables HDV to exploit the same entry receptor, sodium taurocholate co-transporting polypeptide (NTCP), as HBV, facilitating its entry into hepatocytes [11].

Once inside hepatocytes, the uncoated HDV genome moves to the nucleus for ribonucleic acid (RNA)–dependent replication, relying on host deoxyribonucleic acid (DNA)–dependent RNA polymerases I and II [12]. Encoded by the HDV genome, the delta antigen exists in two forms: small (S-HDAg) and large (L-HDAg) delta antigen [13]. RNA editing adds 19 amino acids to the C-terminus of L-HDAg, influencing the functions of both forms. S-HDAg serves as a replication co-factor, while L-HDAg inhibits replication [14]. The farnesylation of L-HDAg is essential for its interaction with HBsAg, anchoring it to the viral envelope and forming a ribonucleoprotein complex critical for the assembly and release of infectious HDV particles [15]. Cells infected with HBV often produce an excess of HBsAg beyond what is required for HBV virion assembly. HDV takes advantage of these surplus empty HBsAg envelopes to coat its ribonucleoprotein complexes [16].

HDV can propagate via cell division, generating clusters of infected cells [17]. Interferon-stimulated genes (ISGs) curtail spread, and lack of interferon (IFN) response enhances HDV propagation [18]. Therapeutic IFN treatment inhibits the mitosis-mediated intrahepatic spread of replicative HDV RNA in HBsAg-positive cells, showing potential therapeutic effects. However, IFN’s limited effects on HDV replication in resting hepatocytes explain its limited clinical efficacy [19].

Targeting HDV is challenging due to its intricate life cycle, HBV dependency, and host polymerase reliance. Still, enhanced HDV virology understanding has unveiled promising therapeutic avenues for control and potential cure, as shown in Fig. 1.

### Natural History of the Disease

#### Transmission

HDV primarily spreads through the parenteral route, via infected body fluids (BF), similar to HBV. Intravenous users (IVDUs) are at highest risk for HDV transmission, but needle exchange programs and increasing vaccination efforts have led to a decline in HDV prevalence among them [20, 21]. Although rare, vertical transmission from an infected mother to the fetus can occur during pregnancy or childbirth [22], but breastfeeding-related transmission has not been reported.

#### Co-infection vs Superinfection

Co-infection refers to simultaneous HDV and HBV infection in individuals with no prior HBV exposure. It can lead to acute self-limiting hepatitis, with most individuals recovering fully [23].

Superinfection occurs when HDV infects an individual with a pre-existing HBV infection. Approximately 90% of superinfection cases are associated with a chronic aggressive disease course leading to increased risk of disease progression, including cirrhosis and hepatocellular carcinoma (HCC) [23]. Serological testing as shown in Table 1 can aid in differentiating acute co-infection and superinfection.

#### Genotypes and Regional Prevalence

Genetically diverse, HDV has eight genotypes and two to four subtypes each [24]. Genotype 1, the most prevalent globally, is associated with aggressive disease and found predominantly in Europe and North America. Genotype 2 is prevalent in Asia, genotype 3 is the most pathogenic and caused fulminant HDV outbreak in the Amazon basin. Genotype 4 (Japan, Taiwan) and genotypes 5–8 (Africa) differ in outcomes, with genotype 5 showing better IFN response [25, 26].

### Underdiagnosis and Impact

Understanding the natural history of HDV has been challenging due to limited prospective studies and data heterogeneity. Prevalence estimates vary, but around 5–14% of HBV patients may have HDV co-infection [3•, 27]. Certain regions are HDV “hotspots,” like Mongolia, Pakistan, Afghanistan, sub-Saharan Africa, Mediterranean, and eastern European countries. Conversely, India, China, Iran, and Israel have declining HDV prevalence (1, 3).

Despite its impact, HDV remains underestimated, particularly in the USA. In a study of 2175 veterans with HBV, merely 8.5% were screened for HDV, and of those tested, 3.4% were HDV-positive. Notably, HDV-positive patients had a 2.9 times higher incidence of HCC compared to those without HDV testing (4). Another study involving 1191 HBV patients revealed that only 8% were tested for HDV, with 73% of the coinfected patients showing cirrhosis, compared to 22% in HBV mono-infection.(5).

### Enhancing HDV Screening

Screening for HDV is crucial for early detection and understanding its natural history. Historically, global HDV screening rates have been low, leading to underdiagnosis, despite its severe health ramifications. In the USA, only 6.7% of HBV patients undergo HDV screening [28]. National Health and Nutrition Examination Survey (NHANES) data revealed about 42% of HBsAg carriers tested positive for anti-HDV, emphasizing the importance of routine HDV screening [29]. This urgency was further emphasized by a Spanish study, which showcased a fivefold increase in HDV diagnoses through universal screening among all HBV patients, advocating for broader screening initiatives [30].

High-risk groups, such as HBsAg-positive individuals, those with human immunodeficiency virus (HIV), IVDU, men who have sex with men, and immigrants from HDV-endemic areas, should be screened according to the American Association for the Study of Liver Diseases (AASLD) (7). Conversely, European and Asian associations advocate for universal HDV testing in all chronic HBV patients due to severity and rising prevalence [31, 32]. A systematic review revealed a pooled HDV co-infection prevalence of 14.57%, with a seroprevalence of 10.58% in individuals lacking risk factors, further endorsing the significance of routine screening over a risk-based approach [33]. Globally, HDV infection risks differ, with higher-income countries experiencing risks from immigration and IVDU [34], while lower-income countries face risks from sexual transmission, intra-household transmission, and cultural practices like tattoos and circumcision (1). Consequently, embracing universal screening emerges as the most effective strategy to gauge HDV’s global impact [35].

### Diagnosis and Staging

#### Diagnostic Algorithm and Challenges in HDV Testing

**Fig. 2:**
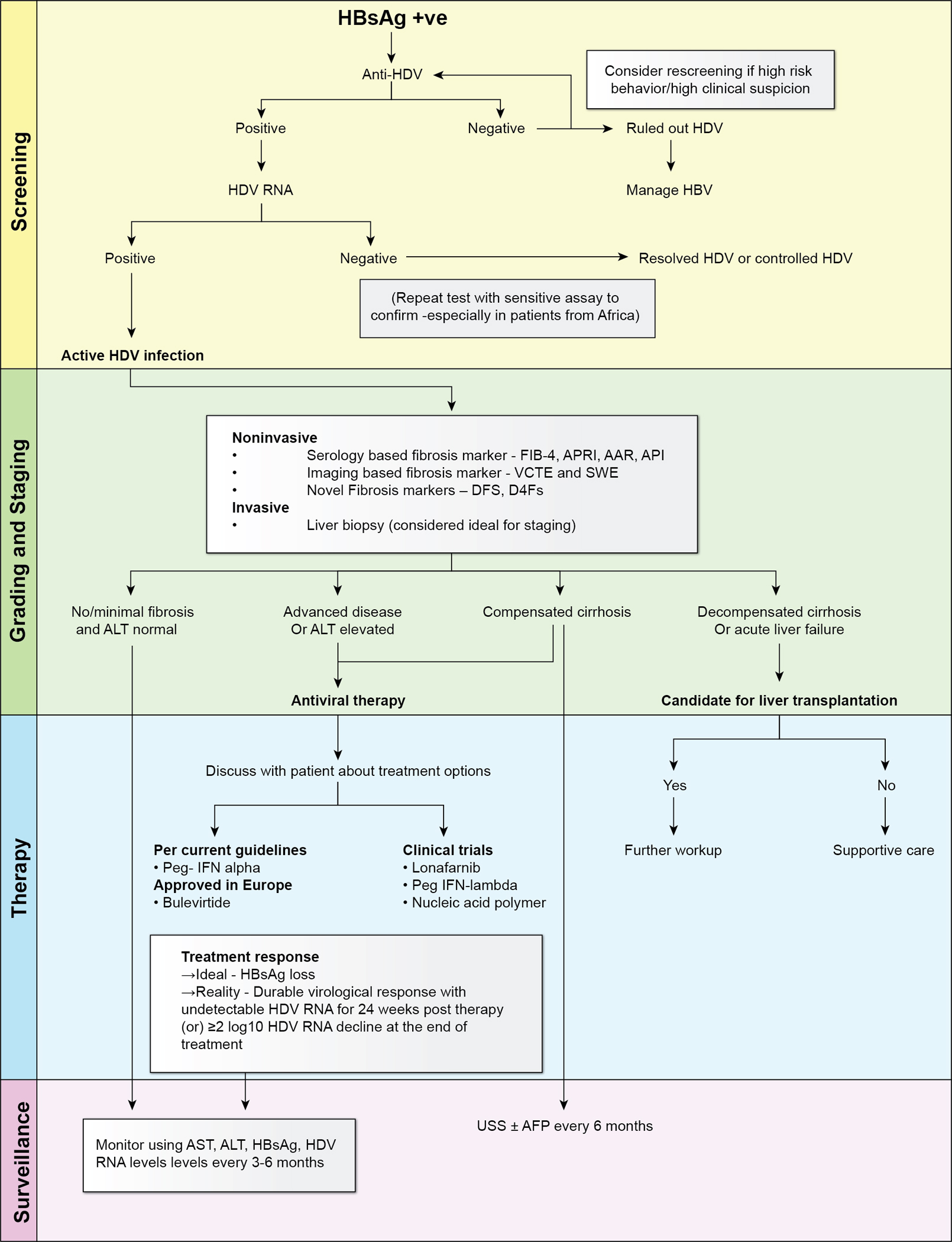
Algorithm for the diagnosis and management of hepatitis D virus

The diagnostic algorithm for HDV starts with screening for the presence of anti-HDV antibodies in patients who are HBsAg-positive [6, 7, 31]. A positive result could indicate prior HDV exposure, necessitating further testing to determine whether the infection is active or resolved (Fig. 2). To confirm active HDV infection, sensitive HDV RNA testing using reverse transcriptase-polymerase chain reaction (RT-PCR) assays is recommended [36]. Several assays have evolved with heightened sensitivity, detecting HDV RNA levels as low as 6 international units (IU) per milliliter. However, it is crucial to emphasize the utilization of validated commercial assays due to variations in detection among different assays and the need for standardized HDV RNA measurement across laboratories [37, 38].

The first World Health Organization international standard for HDV RNA was established in 2013, based on a genotype 1 HDV-infected patient. Despite its availability, in-house nucleic acid amplification testing protocols are commonly used for HDV RNA quantification. Automated isolation may underestimate viral load, and certain genotypes exhibit quantification errors [39, 40]. One other challenge that exists is diagnostic variability in anti-HDV serology assays due to factors like assay setup, antigen type (recombinant HDAg, peptides), and interference from other materials (serum proteins). Improved HDV diagnostic serology requires automated, reproducible assays with high diagnostic accuracy and quantification capability for better reliability [41]. Newly developed droplet digital PCR and cobas6800 offer improved accuracy with low limits of detection, promising enhanced HDV RNA quantification [42].

## Staging Methods

Once HDV is confirmed, grading and staging become pivotal for assessing disease severity and progression. While liver biopsy has historically been the gold standard for staging, noninvasive alternatives have emerged [6, 43].

As shown in Fig. 2, serum fibrosis markers including fibrosis-4 score (FIB-4), aspartate aminotransferase (AST)-to-alanine aminotransferase (ALT) ratio, age-platelet index, and AST-to-platelet-ratio-index can help in predicting fibrosis and cirrhosis progression. Among these, FIB-4 stands out for its performance with AUROC of 0.7 for advanced fibrosis and 0.83 for cirrhosis in HDV, but its performance is relatively lower compared to HCV and HBV [43]. Despite certain limitations, these cost-effective markers hold practical applicability, particularly in resource-limited settings burdened by HDV (Fig. 2).

Vibration-controlled transient elastography (VCTE) and shear wave elastography (SWE) are promising non-invasive imaging modalities for fibrosis assessment in chronic hepatitis D [44]. VCTE is a notable noninvasive method that has demonstrated promising accuracy in cirrhosis detection, notably with a cutoff of 14 kPa [45]. However, further validation through larger studies is warranted to solidify its clinical utility. VCTE, although effective, faces limitations in accessibility due to cost and availability. In contrast, SWE’s global application potential is augmented by its compatibility with existing ultrasound machines through software additions.

### Novel Fibrosis Scoring System

The Delta Fibrosis Score (DFS) and Delta-4 Fibrosis Score (D4FS) have been developed as non-invasive fibrosis scores for HDV. DFS incorporates patient age, albumin, gamma-glutamyl transferase, and cholinesterase (CHE) levels to predict advanced fibrosis, exhibiting good performance with an AUROC of 0.87 [46]. D4FS, which integrates serum fibrosis markers and VCTE, is a better predictor of cirrhosis with an impressive AUROC of 0.94 [47]. Nevertheless, the clinical application of DFS score might be hampered by marker availability, as seen in DFS with CHE. Larger prospective studies are needed to validate and establish the clinical utility of these scores in managing HDV patients.

### Treatment

#### Who Needs to be Treated?

Ideally, every HDV-infected individual should receive treatment, particularly considering the deadly nature of the infection. Nonetheless, due to the clear link between HDV viremia and worsened outcomes, highlighted by a Swedish study indicating a 3.8-fold higher risk of liver-related complications due to HDV RNA viremia over 6.5 years, strategic measures are advised [48]. Accordingly, the AASLD prioritizes treatment for those with elevated HDV RNA levels and ALT elevation (7). Elevated HDV RNA levels frequently correspond to more severe disease manifestations [49]. Conversely, the absence or substantial reduction of HDV RNA aligns with improved clinical outcomes. Emphasizing the pressing requirement for effective treatment strategies, the objective is to manage HDV infection and alleviate its adverse impact.

### Definition of Treatment Response: Ideal vs Reality

Defining effective endpoints in HDV therapy is complex due to the dual challenge of suppressing both HBV and HDV. Proposed surrogate endpoints, like HBsAg clearance and HDV RNA below the lower limit of quantification (LLOQ), correlate with improved clinical outcomes. Sustained virological response (SVR), characterized by undetectable HDV RNA for 24 weeks post-therapy, reduces adverse events during long-term follow-up [50]. However, achieving SVR at 24 weeks remains challenging, as evidenced by relapses observed even after its attainment [51].

An alternative endpoint, proposed by Yurdaydin et al., suggests a ≥ 2 log10 HDV RNA decline at the end of treatment, though this should be labeled a partial response [52, 53••]. Regulatory agencies in the US and Europe have accepted this proposal, acknowledging its potential significance. However, any presence of HDV is associated with increased risks compared to undetectable HDV RNA, casting doubt on the current endpoint’s validity [50, 54]. A study from Spain further questions the endpoint’s relevance by revealing no significant clinical outcome differences between those with ≥ 2 log10 HDV-RNA decline and those without [55]. But patients in this study achieved SVR spontaneously.

As per AASLD-EASL guidelines, for finite therapies, the ideal endpoint involves HBsAg loss, anti-HB seroconversion, and HDV RNA < LLOQ. An alternate goal is HDV RNA < LLOQ at 24 weeks post-treatment, with prolonged follow-up. For maintenance therapy, endpoint is HDV RNA < LLOQ at 48 weeks on-treatment, although optimal duration remains uncertain. Reminiscent of predicting duration of anti-viral treatment for hepatitis C needed to reach < 1 virus copy in a patient’s total extracellular BF [56], Shekhtman et al. [57] predicted time to reach < 1 HDV copy per BF in two patients who were treated with bulevirtide monotherapy for 144 weeks [58]. One of these two patients remained HDV RNA undetectable 72 weeks after bulevirtide discontinuation, suggesting HDV viral cure [59]. If HDV RNA < LLOQ is unattainable, an alternative endpoint is a combined response: > 2 log HDV RNA reduction and normal ALT at 48 weeks, with subsequent follow-up [53••]. These evolving endpoints reflect ongoing efforts to effectively manage HDV infection’s intricate dynamics.

### Treatment Strategies

#### Interferon Alpha

Current guidelines recommend pegylated formulations of interferon alpha (PEG-IFNα) as a primary treatment for HDV [60]. While PEG-IFNα’s 48-week regimen offers convenience and acceptable response rates, complete HDV eradication remains modest [61]. Post-treatment, only a subset of patients (ranging from 23 to 57%) achieve undetectable HDV RNA levels at 24 weeks, often accompanied by high relapse rates [51], especially in HBsAg-positive cases. Undetectable HDV RNA level at 24 weeks off treatment does not universally correlate with HDV clearance, necessitating extended follow-up [51].

A Belgian long-term follow-up study demonstrated SVR in 52% of interferon alpha (IFNα)–treated patients at 24 weeks, with 33% patients relapsing over 3 years. Prolonging treatment beyond 48 weeks minimally impacted durable off-treatment virological response [62]. Importantly, responders to interferon exhibit reduced mortality rates compared to non-responders, emphasizing its clinical significance. A German study with mean follow up of 5.2 years demonstrated that IFNα treatment yields better long-term outcomes than untreated or nucleotide analog-receiving patients [63].

### Interferon Lambda

Interferon lambda (IFN-λ) offers a promising avenue for HDV treatment, thanks to its selective hepatocyte receptor expression and reduced side effect profile. A phase II trial explored two doses (120 g and 180 g) of IFN-λ for 48 weeks and resulted in viral load below limit of quantification (BLQ) in 16% and 36% of patients in the respective arms at 24 weeks post-treatment. The 180-g arm demonstrated comparable SVR to PEG-IFNα, with fewer side effects [64].

### Bulevirtide

HDV exploits the NTCP receptor, similar to HBV, for hepatocyte entry. Bulevirtide (myrcludex B), is a synthetic myristoylated peptide that binds to NTCP receptors, disrupting viral entry. In the MYR-202 trial with 120 HDV patients, different bulevirtide doses with tenofovir combinations showed significant HDV RNA reduction (50–77%) compared to tenofovir alone (4%). However, bulevirtide had minimal impact on HBsAg levels, and discontinuation led to HDV RNA rebound [65]. In a phase 3 MYR-301 trial, patients received subcutaneous bulevirtide (2 mg or 10 mg daily) or no treatment for 144 weeks. The 48-week interim analysis showed a higher response rate (45% in 2 mg, 48% in 10 mg) than controls (2%), meeting the primary endpoint of undetectable HDV RNA or significant decrease with ALT normalization. Yet only 12% in 2 mg and 20% in 10 mg groups achieved undetectable HDV RNA at week 48. HBsAg levels did not significantly decrease in either bulevirtide group (8). Real-world data demonstrated a 76% virologic response (≥ 2 log HDV RNA decline or undetectable) with bulevirtide, but relapse occurred in some cases, and no patients lost HBsAg [66].

### Combining Bulevirtide with Peginterferon

The MYR-203 trial examined bulevirtide alone or with PEG-IFNα or tenofovir in 60 patients across four arms for 48 weeks. Low-dose bulevirtide with PEG-IFNα and high-dose bulevirtide with PEG-IFNα maintained undetectable HDV RNA levels in 8/15 and 4/15 patients, respectively [67]. MYR-204, a phase 2b study, demonstrated higher HDV-RNA decline rates in combination arms compared to PEG-IFNα monotherapy [68].

Despite bulevirtide showing promise, its long-term use, discontinuation viability, safety in decompensated cirrhotic patients, and potential dose-related bile acid increase will require further exploration.

### Nucleic Acid Polymer

Nucleic acid polymers targeting host chaperones have shown potential in disrupting HBsAg assembly and release. One promising candidate is REP 2139-Ca. In a small study, patients received REP 2139-Ca monotherapy for 15 weeks, followed by PEG-IFNα for an additional 15 weeks, and then PEG-IFNα monotherapy for 33 weeks. Astonishingly, seven of 12 patients achieved undetectable HDV RNA levels, and five had HBsAg loss. Impressively, a 3.5-year follow-up demonstrated sustained outcomes, with seven of 11 patients maintaining undetectable HDV RNA levels and four achieving HBsAg loss [69, 70]. Mathematical modeling indicated that the median efficacy of REP 2139-Ca monotherapy in blocking HBsAg and HDV production was 98.2% and 99.7%, respectively. In addition, a short HBsAg half-life of 1.3 days was estimated, suggesting a rapid turnover of HBsAg in HBV/HDV co-infected patient [71•]. To validate these findings, large-scale studies are imperative.

### Lonafarnib

Lonafarnib, a farnesyl-transferase inhibitor, interferes with HDV assembly and secretion of HDV virions [72]. Mathematical modeling indicated high efficacy of lonafarnib in blocking HDV production (95%) along with an estimated HDV half-life of 1.6 days. While initial lonafarnib monotherapy studies reduced HDV viral load, they resulted in elevated gastrointestinal adverse events. However, combining low-dose lonafarnib (25 or 50 mg twice daily) with the cytochrome P450 3A4 inhibitor ritonavir proved beneficial, yielding favorable antiviral effects with reduced side effects. Notably, lonafarnib 25 mg with ritonavir + PEG-IFNα achieved a remarkable mean log decline of − 5.57 log10 U/ml in HDV RNA levels, with 60% of patients achieving undetectable HDV-RNA and ALT normalization [73].

### Combining Lonafarnib with Peginterferon

The preliminary data from recently concluded LIFT study explored triple therapy with peginterferon lambda (PEG-IFN-λ), lonafarnib, and ritonavir in HDV patients for 24 weeks. After 12 weeks, mean HDV RNA levels dropped by 3.4 log IU/mL. At therapy conclusion, the mean decline was 3.2 log IU/mL (50% undetectable or BLQ). Impressively, 96% achieved a > 2 log decline during the 24-week treatment period, accompanied by mostly mild to moderate adverse events [74•]. The encouraging LIFT study outcomes set the stage for an upcoming 48-week trial, exploring the extended efficacy of triple combination therapy.(ClinicalTrials.gov NCT05953545). This extension holds potential for further enhancing positive results.

In the phase 3 D-LIVR study with 407 patients, ritonavir-boosted lonafarnib alone or combined with PEG-IFNα was evaluated for 48 weeks. Combination therapy (19.2%) showed superior response rates compared to lonafarnib/ritonavir (10.1%) or PEG-IFNα alone (9.6%) in achieving the primary endpoint of ≥ 2 log HDV RNA reduction with ALT normalization [75].

### Role of Nucleos(t)Ide Analogues in HDV Management

While nucleos(t)ide analogues (NAs) alone may not achieve HDV cure [76], they still might have a role to play and are frequently used alongside HDV treatment due to potential HBV reactivation after HDV control [72]. This requires further clarification. Additionally, the role of NA in compensated HBV/HDV cirrhosis with low HBV DNA levels needs investigation.

### Monitoring Response to Treatment and Disease Progression

The study conducted by Guedj et al. has yielded a significant breakthrough, shedding light on the intricate dynamics of early HDV and HBsAg kinetics during PEG-IFNα therapy. Through a novel mathematical model, this research offers crucial insights into the complex interplay among HDV, the host, interferon treatment impact, and treatment response predictors. Analyzing comprehensive HDV and HBsAg kinetic data over 28 weeks from 10 PEG-IFNα-treated patients, the study revealed a biphasic decline in HDV levels—a rapid initial phase followed by a slower or plateau phase. Impressively, PEG-IFNα’s efficacy in suppressing HDV production was high, with a median effectiveness of 96%. The correlation between HBsAg kinetics and the latter phase of HDV decline highlighted the role of HBsAgproductive–infected cells in HDV production. Clinically, this finding is notable, particularly in identifying a flat second phase in HDV and HBsAg kinetics as a potential predictive marker for incomplete virological response during PEG-IFNα therapy. This discovery holds promise for shaping early discontinuation criteria, optimizing decision-making in HDV management [77].

Another study explored the role of HBsAg in HDV-RNA clearance during PEG-IFNα treatment. A reduction in HBsAg below 1000 IU/mL at the 6 months indicated favorable outcomes. A 0.105 log HBsAg reduction correlated with 1.610 log HDV-RNA reduction at 6 months on treatment, predicting HDV clearance [78]. Hence, quantitative HBsAg could guide therapy decisions. Hepatitis B core-related antigen (HBcrAg) linked to intrahepatic covalently closed circular DNA levels can aid in predicting treatment response. In a study which investigated this, baseline HBcrAg < 4.5 log IU/ml increased chances of achieving undetectable HDV RNA at 24-week follow-up (NPV 81.8%), with higher endof-treatment HBcrAg linked to a higher relapse rate [79].

### Screening for Hepatocellular Carcinoma

The intricate relationship between HDV and HBV, where HDV relies on HBV for transmission, adds complexity to understanding HDV’s role in hepatocarcinogenesis. In Italy, a long-term study estimated annual incidences of liver decompensation in HDV cirrhosis at 4%, with HCC incidence at 2.8% [54]. Patients with HDV cirrhosis are at threefold higher risk of developing HCC than those with HBV mono-infection associated cirrhosis [80], emphasizing importance of strict screening in those with HDV infection.

Interestingly, in non-cirrhotic patients, HDV replication increased the likelihood of progression to HCC even in absence of cirrhosis [81]. However, uncertainties persist regarding whether individuals without cirrhosis should undergo HCC screening and the appropriate age to initiate screening for those without cirrhosis. A study from Mongolia showed that HBV-HDV coinfection contributes to HCC development at a comparatively younger age in contrast to those infected with hepatitis C virus (mean age 51 vs 59). However, this study did not differentiate between cirrhotic and non-cirrhotic patients [82].

These findings emphasize the relationship between HDV and HCC, suggesting the need for vigilant HCC screening strategies, especially in HDV-infected cirrhotic. Further research is required to delineate optimal screening protocols and the age at which to start HCC surveillance in non-cirrhotic HDV patients.

### Liver Transplantation

In HDV patients with decompensated cirrhosis or fulminant hepatitis, the use of interferon is restricted due to adverse effects and limited data. Consequently, liver transplantation (LT) remains the primary treatment avenue [83]. In one of the most extensive studies involving 76 LT in HDV patients, a remarkable 88% survival rate was observed for 5 years follow-up [84].

Historically, LT for HDV-induced cirrhosis has yielded favorable clinical outcomes, although accompanied by significant graft reinfection rates (70–80%) [85]. As expected, the presence of HBV viremia proved pivotal for HDV recurrence (1). The introduction of prolonged hepatitis B immunoglobulin (HBIg) prophylaxis successfully lowered HDV reinfection rates and further improved through HBIg and NA combinations [86]. A comprehensive European cohort study revealed a mere 3.5% HBV recurrence post-LT, with negligible HDV recurrences [87].

Recent studies investigate stopping HBIg with ongoing NAs after HDV-related transplants, noting minimal HDV reinfections, validating efficacy of NAs alone in controlling relapse [88, 89]. Collectively, prophylactic approaches have substantially diminished HDV recurrence rates post-LT, offering optimism for enhanced patient outcomes.

### HBV Vaccination to Reduce HDV

The absence of an HDV vaccine underscores the significance of HBV vaccination as a key strategy for preventing HBV and thus potentially curtailing HDV transmission. The implementation of HBV vaccination has led to a decline in HBV infections, consequently protecting high-risk groups from acquiring HDV infection [90]. This is evidenced by the age-related shift in HDV prevalence towards older individuals, reflecting the success of HBV vaccination programs in preventing early HBV infections [91]. By effectively preventing HBV, vaccination indirectly contributes to the reduction of HDV transmission, offering long-term protection against these viral infections.

### Critical Questions

In the realm of HDV infection management, several pivotal questions arise. What is the true prevalence of HDV? What drives the swift advancement to cirrhosis and hepatocellular carcinoma in HDV-infected individuals? As we seek meaningful treatment endpoints, does a 2-log decline in HDV RNA levels truly correlate with improved clinical outcomes, and are there alternative endpoints which offer valuable insights? The optimal treatment duration for each HDV agent warrants consideration, as does the potential synergy of combination therapies. Amid these complexities, the feasibility of treating individuals with decompensated cirrhosis remains a critical query. Shifting focus, determining the appropriate age to initiate HCC screening in HDV-infected patients and investigating possible associations between genotypes and disease progression add further layers of inquiry. Lastly, the question of reactivation or relapse in those who have cleared HDV infection looms, demanding vigilant understanding. Addressing these questions are important in advancing comprehension of HDV infection management.

## Conclusions

Addressing the complex challenges posed by HDV infection requires a multi-faceted approach. Raising awareness among healthcare professionals is imperative, advocating for reflex screening of HBV patients for anti-HDV antibodies. In cases of positivity, a subsequent step involves using a reliable and validated quantitative HDV RNA assays to confirm active infection. Timely and accurate assessment, facilitated by validated serological biomarkers and non-invasive tests, is crucial for effective diagnosis and risk stratification. Immunization against HBV stands as a potent preventive measure. While financial constraints and awareness gaps persist, ongoing research and innovative therapies fuel hope for better outcomes. Understanding viral-host-drug dynamics may help to develop and optimize response-guided therapies for HDV patients. This dedication to understanding and treating HDV brings hope for millions suffering from this deadly infection, promising enhanced global health in the face of its enduring challenges.

## Figures and Tables

**Table 1:** Serological markers to assess for HBV and HDV infection

	ALT	HBsAg	Anti HBsAg	Anti-HBc Ag IgM	Anti-HBc Ag IgG	HBeAg	Anti-HBeAg	HBV DNA	Anti-HDV ab	HDV RNA	HDV Ag

Acute HBV/HDV co-infection	↑↑	+	−	+	−/ +	−/ +	−	+	+	+	+
Acute HBV/HDV superinfection	↑↑	+	−	−	+	±	−/ +	+	+	+	±
Chronic HBV/HDV infection	↑	+	−	−	+	±	−/ +	+	+	+	−
Resolved HDV infection	↑ or↔	+	−	−	+	±	−/ +	+	+	−	−
Resolved both HBV/HDV infection	−	−	±	−	+	−	+	−	+	−	−
False positive	−	−	−	−	−	−	−	−	+	−	−
False negative	↑ or ↔	+	−	−	+	±	−/ +	+	−	+	−

*ALT* alanine aminotransferase, *HBsAg* hepatitis B surface antigen, *HBeAg* hepatitis B e Antigen, *HBc* hepatitis B core, *IgG* immunoglobulin G, *IgM* immunoglobulin M, *HBV* hepatitis B virus, HDV hepatitis D virus

* HBV RNA and hepatitis B core antigen (HBcrAg) are serological markers that show promise in predicting treatment response but need further validation

## Abbreviations

AAR: AST-to-ALT ratio
AASLD: American Association for the Study of Liver Disease
ALT: Alanine aminotransferase
API: Age platelet index
APRI: AST-to-platelet ratio index
AST: Aspartate aminotransferase
AUROC: Area under the receiver operating characteristic curve
BF: Body fluid
CHB: Chronic hepatitis B
CHC: Chronic hepatitis C
CHD: Chronic hepatitis D
CHE: Cholinesterase
CSPH: Clinically significant portal hypertension
D4FS: Delta-4 fibrosis score
DFS: Delta fibrosis score
DNA: Deoxyribonucleic acid
EASL: European Association for the Study of the Liver
ELISA: Enzyme-linked immunosorbent assay
FIB-4: Fibrosis-4
FDA: Food and Drug Administration
GGT: Gamma glutamyl transferase
HBcrAg: Hepatitis B core antigen
HBeAg: Hepatitis B e antigen
HBsAg: Hepatitis B surface antigen
HBV: Hepatitis B virus
HBIg: Hepatitis B immunoglobulin
HDAg: Hepatitis D antigen
HDV: Hepatitis delta virus
HCC: Hepatocellular carcinoma
HIV: Human immunodeficiency virus
IU: International units
IVDU: Intravenous drug users
kPa: Kilopascals
LLOQ: Lower limit of quantification
LT: Liver transplantation
NHANES: National Health and Nutrition Examination Survey
NPV: Negative predictive value
NTCP: Sodium taurocholate cotransporting polypeptide
RT-PCR: Reverse transcriptase-polymerase chain reaction
PEG-IFN: Pegylated interferon
PCR: Polymerase chain reaction
RNA: Ribonucleic acid
SWE: Shear wave elastography
VCTE: Vibration-controlled transient elastography
